# Geographical Patterns and Drivers of Species and Phylogenetic Diversity of Desert Plant Communities in the Hexi Corridor, Northwestern China

**DOI:** 10.1002/ece3.72114

**Published:** 2025-09-07

**Authors:** Xinyi Zhou, Zhaoxiang Zhang, James F. White, Yingxiang Miao, Shanjia Li

**Affiliations:** ^1^ School of Life Science and Engineering Lanzhou University of Technology Lanzhou China; ^2^ Department of Plant Biology Rutgers University New Brunswick New Jersey USA; ^3^ Key Laboratory of Land Surface Process and Climate Change in Cold and Arid Regions Northwest Institute of Eco‐Environment and Resources, Chinese Academy of Sciences Lanzhou China

**Keywords:** desert plant communities, driving factors, geographical patterns, phylogenetic diversity, species diversity

## Abstract

Desert plant communities play an irreplaceable role in maintaining the ecological balance of arid areas. Understanding the spatial distribution pattern of desert plant diversity and its environmental response mechanism is particularly important for the protection of regional biodiversity, and combining phylogenetic information can provide more in‐depth insights. To this end, this study conducted a survey of desert plant communities along the southeast to northwest direction of the Hexi Corridor, revealing the variation patterns of species and phylogenetic diversity (PD) indicators along longitude, latitude, and altitude, and explored the driving factors of these patterns in combination with geographical, climatic, and soil factors. The results showed that the changes in species diversity (Shannon–Wiener and Margalef) and PD along longitude and latitude showed a unimodal model, with the highest value in the central region. The dispersion of phylogenetic structure decreases with increasing altitude, with divergent patterns at low altitudes and clustered patterns at high altitudes. In addition, we found that soil factors such as soil available phosphorus (SAP), soil total phosphorus (STP), and soil available nitrogen (SAN) dominated the variation of species diversity, and the PD was also mainly regulated by soil available phosphorus (SAP), while the main influencing factor of the phylogenetic structure was the average annual temperature (AMT), indicating that the community diversity pattern was driven by soil nutrients and climatic factors. The study reveals the relative roles of different environmental factors in shaping community diversity and provides a scientific basis for formulating effective desert ecosystem protection strategies.

## Graphical abstract

This study analyzes the spatial distribution and driving factors of plant species and phylogenetic diversity in Hexi Corridor, and points out that the central region has the highest diversity and that the climate and soil jointly regulate the diversity pattern, which is beneficial to the formulation of conservation strategies in desert areas.

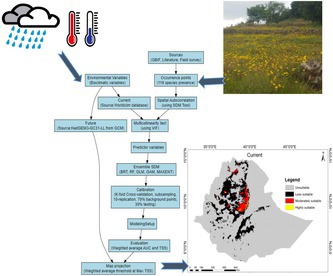

## 1 Introduction

Desert ecosystems play a significant role in maintaining soil stability, preventing erosion, and preserving unique biodiversity, holding a distinctive and vital position within global ecosystems (Wang et al. 2019). However, these ecosystems are typically constrained by limited water and nutrient availability, exhibiting sparse vegetation with pronounced patchy distribution patterns, rendering them more fragile compared to other ecosystems (Elser 2000). Compounded by climate change and intensifying drought, desert regions face escalating challenges such as declining plant diversity and accelerated nutrient depletion (Tai et al. 2021). Therefore, it is crucial to conduct in‐depth research on the characteristics of desert plant communities and their diversity distribution patterns, and to analyze their environmental adaptation mechanisms.

The geographical pattern of biological distribution has always been a key research direction (Willis and Whittaker 2002; Ricklefs 2004). Understanding the geographical distribution pattern of desert plants is helpful to reveal the laws of biodiversity change and specify conservation strategies. Geographical factors such as longitude, latitude, and altitude have a significant impact on plant diversity. These factors jointly shape the distribution pattern of plant communities by affecting water, energy, and soil conditions (Zhang et al. 2024). For example, on a global scale, plant species richness generally decreases with increasing latitude, decreasing from the tropics to the poles (Willig et al. 2003). However, due to the complex influence of geographical and environmental factors and their interactions, the spatial distribution pattern of plant diversity varies in different research areas and at different scales (Pan et al. 2023). According to previous studies, the distribution pattern of plant diversity along geographical gradients is mainly linear (da Silva et al. 2014), unimodal (Zhang and Zhang 2017), or uncorrelated (Wang et al. 2016) patterns. For example, in the Helan Mountain Nature Reserve in Ningxia, community diversity showed a unimodal pattern along the altitude gradient (He et al. 2023); while research in Turpan, Xinjiang, showed that plant richness was positively linearly correlated with longitude and altitude (Tu et al. 2024). In contrast, large‐scale studies conducted in the desert areas of northwest China showed that plant richness first decreased and then increased along the longitude and latitude gradients, but no clear pattern was found between plant richness and altitude gradients (Jianming et al. 2017). Therefore, the geographical distribution pattern of plant diversity needs to be analyzed specifically based on the research area and habitat factors.

Environmental factors such as precipitation, temperature, and soil nutrients are closely related to the distribution pattern of plant diversity. Changes in precipitation can affect the pattern of species richness and species composition (Báez et al. 2013), ecosystem net primary productivity (Heisler‐White et al. 2009), and carbon cycle (Harper et al. 2005). Desert ecosystems, which are strictly restricted by water, respond particularly well to precipitation fluctuations (Tan et al. 2021). Most studies generally believe that water regulates community construction through resource supply. Increased precipitation is beneficial to plant growth and increases species diversity, while drought inhibits species coexistence (Xiao et al. 2023; Griffin‐Nolan et al. 2019; Liu et al. 2018). However, the latest research has found that moderate drought may improve the species diversity of communities by alleviating the resource competition pressure of dominant species and promoting the niche differentiation of rare species (Alon and Sternberg 2019). As for temperature, rising temperatures in arid regions have exacerbated water shortages and changed physiological processes such as plant photosynthesis, thereby affecting species distribution and community composition (Sardans et al. 2024; Sharaya et al. 2023). In addition, temperature also interacts with other environmental variables to jointly regulate plant diversity (Wangchuk et al. 2021). Soil, as a plant growth medium, provides plants with a large amount of nutrients and water (Zuo et al. 2012). A large number of studies have shown that soil organic matter, nitrogen, and phosphorus have significant effects on plant richness and community composition (Becknell and Powers 2014; Hu et al. 2021). As soil nutrient levels increase along the environmental gradient, ecosystems may accommodate more species, thereby increasing their diversity (Oehri et al. 2017). In addition, in semi‐arid and dryland ecosystems, changes in soil physical and chemical characteristics under water stress conditions can significantly regulate the competitive dynamics and mutualistic relationships between plant species (Fowler 1986). However, most previous studies have focused on the impact of a single environmental factor on plant diversity and rarely considered these environmental factors and their relative contributions (Jianming et al. 2017; Wani et al. 2025). In addition, most studies have focused on species‐rich hotspots, and research on desert ecosystems is clearly insufficient. Therefore, it is crucial to comprehensively analyze the impact of soil and climate factors on desert plant diversity and identify the key driving factors.

Most studies of plant diversity have only explored species diversity. However, this approach has some limitations as they ignore the evolutionary relationships between species, which may lead to underrepresentation of interspecific differences (Jia and Du 2014). Phylogenetic diversity (PD), as an alternative biodiversity measure, can reveal the evolutionary history among different species and analyze the current status and causes of community species composition from an evolutionary perspective (Wicke and Fischer 2017; Cadotte et al. 2009). Phylogenetic structure provides an important perspective for analyzing the mechanism of community construction, and its formation mechanism is often explained by niche theory and neutral theory (Kelly et al. 2008). Niche theory holds that, based on the premise of regional plant niche conservation, habitat filtering and competitive exclusion jointly regulate community species composition. When resources are limited and environmental filtering plays a dominant role in the community, closely related species end up together. In contrast, when resources are abundant and competitive exclusion plays a dominant role in the community, coexisting species tend to be distantly related species (Webb and Donoghue 2005; Webb et al. 2002). Neutral theory holds that all species are equivalent in niche. The species composition of local communities is mainly derived from the dynamic balance of species with similar ecological functions in the process of diffusion, colonization, and random extinction. Its species replacement pattern is more of a random rather than a deterministic process, which is mainly related to diffusion restriction (Sproull et al. 2015). In addition, key environmental factors driving species diversity versus PD may also differ in the same regional study (Liang et al. 2023). Therefore, in plant diversity studies, it is necessary to integrate PD for analysis in addition to traditional species diversity indicators, which is more conducive to revealing community succession mechanisms, interspecific patterns, and ecosystem function maintenance mechanisms.

The Hexi Corridor is an important passage of the Silk Road in northern China. Its vegetation community is mainly composed of a large number of drought‐resistant desert plants, which is a typical desert ecosystem (Zhang, Li, et al. 2020). The Hexi Corridor has low precipitation, large evapotranspiration, scarce vegetation cover, and severe soil erosion (Su et al. 2007). Its unique desert environment provides a natural laboratory for exploring ecological adaptation mechanisms under extreme conditions. In addition, the Hexi Corridor extends from southeast to northwest, forming a significant environmental gradient, providing an ideal natural experimental field for scientific research. However, there is no complete study on the species and phylogenetic patterns of the desert plant communities in the Hexi Corridor.

In this study, we conducted community surveys along the southeast to northwest direction of the Hexi Corridor and analyzed the variation patterns of plant species diversity and PD along longitude, latitude, and altitude. We also used key environmental factors such as geography, temperature, precipitation, and soil nutrients to explain the spatial variation patterns of plant diversity. Specifically, we explored the following questions: (1) What is the pattern of the geographical distribution of species diversity and PD in the region? (2) How do geography, climate, and soil affect the diversity of these two dimensions? (3) Which factors have the most important impact on plant diversity? This study helps to reveal the evolutionary laws of regional ecosystems and can also provide certain scientific insights for desertification prevention, ecological protection, and biodiversity maintenance in the context of climate change in the Hexi Corridor.

## 2 Materials and Methods

### 2.1 Study Area

**FIGURE 1:**
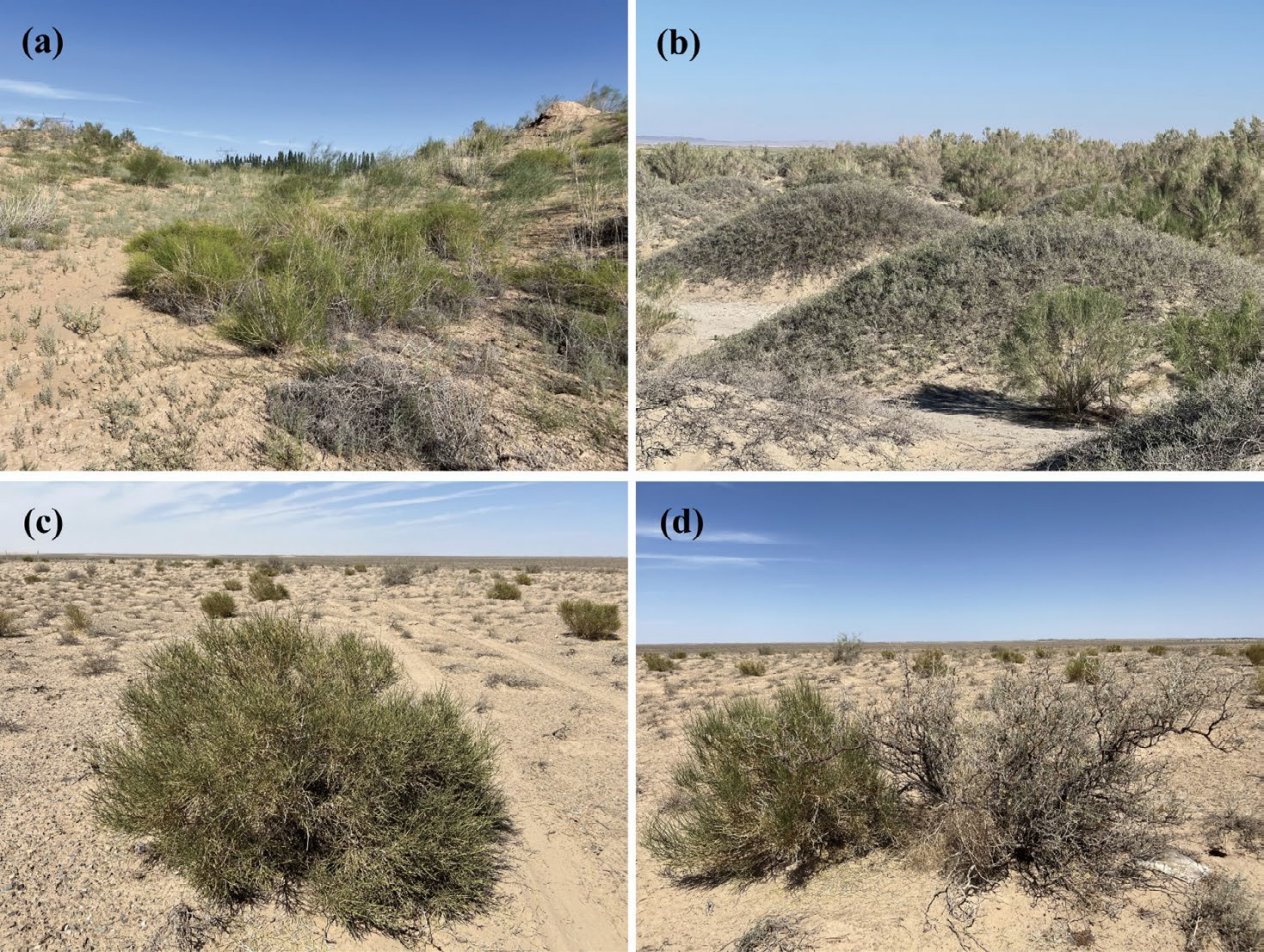
Photographs of typical sampling plots and desert plants in the Hexi Corridor. (a) Eastern part of typical sampling plots (HX1–4); (b) middle part of typical sampling plots (HX5‐10); (c, d) western part of typical sampling plots (HX11–16).

The Hexi Corridor is located in Gansu Province in northwestern China. It runs from southeast to northwest. The eastern section starts at Wushaoling Mountains, and the western section ends at Yumenguan. Its longitude ranges from 92°21′ to 104°45′ E, and its latitude ranges from 37°15′ to 41°30′ N. The region has a typical temperate continental climate, with an average annual temperature of 5°C–9°C, an average annual sunshine of 2800–3300 h, an annual evaporation range of 1500–3200 mm, and an annual precipitation range of 50–300 mm, which decreases from southeast to northwest. The vegetation in the Hexi Corridor is sparse and unevenly distributed, mainly composed of drought‐tolerant shrubs, subshrubs, and herbs. Some typical desert plant communities are shown in Figure 1. The soil has low water content and very little surface humus and is mainly composed of gravel, sand, and clay (Zhang and Zhao 2015).

### 2.2 Plot Setting and Sampling

**FIGURE 2:**
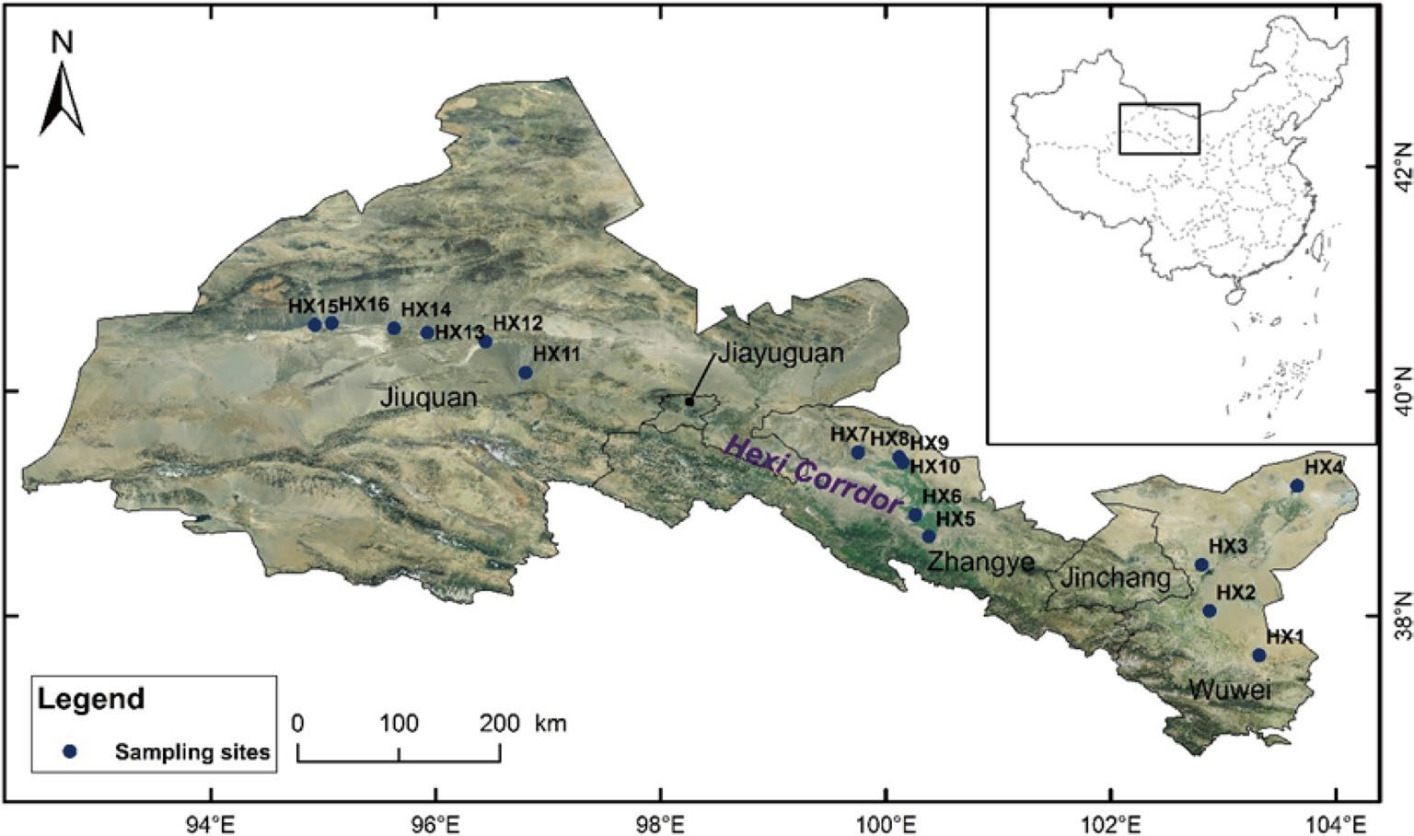
Survey routes and sample plot distribution of desert plant communities in the Hexi Corridor, China.

A total of 16 fixed transects (HX1–HX16, Figure 2) were set up on the gradient of natural precipitation gradually decreasing from the southeast to the northwest of the Hexi Corridor. Three 20 m × 20 m survey quadrats were set up in each transect, each separated by 10 m. All quadrats were in a natural state without human interference. In each quadrat, three 5 m × 5 m plots were randomly set up for shrub community surveys, and three 2 m × 2 m small plots were set up for herbaceous plant community surveys. The basic information of the transects (such as longitude and latitude, altitude, etc.) and plant community characteristics, including plant species, number of individuals, height, canopy width, and coverage, were recorded. The sampling period was from July to September 2024, which is the peak growth season for desert plants. Plant species identification was based on the Flora of China. In addition, three to five 0–20 cm soil profiles were randomly selected in each transect to collect soil samples, which were mixed and stored in sealed bags to avoid the mixing of plant litter.

### 2.3 Acquisition of Environmental Factors

The GPS system of the smartphone was used to record the latitude, longitude, and altitude of the sampling points. In order to obtain climate factors, we used ArcGIS 10.5 software to extract climate factor data with a spatial resolution of 30 arc sec for each sample strip from the WorldClim database (Fick and Hijmans 2017). Climate factors include annual mean temperature (AMT), maximum temperature of the warmest month (MTWM), minimum temperature of the coldest month (MTCM), and annual precipitation (AP). In addition, we measured soil organic carbon (SOC), soil available phosphorus (SAP), soil available nitrogen (SAN), soil total phosphorus (STP), soil total nitrogen (STN), and soil pH (SpH) in the laboratory as soil factor indicators. Soil organic carbon was determined by potassium dichromate oxidation method, and soil total nitrogen was determined by automatic Kjeldahl nitrogen analyzer; soil total phosphorus and soil available phosphorus were determined by molybdenum antimony method; soil available nitrogen was determined by alkaline solution diffusion method; soil pH was determined by PHS‐3D pH meter produced by Shanghai Precision Scientific Instrument Co. Ltd. (Li et al. 2021).

### 2.4 Selection and Quantification of Diversity Indicators

Three indices were used to assess species diversity, including the Shannon–Weiner diversity index (*H*), the Pielou evenness index (*E*), and the Margalef richness index (*S*), which take into account the richness and evenness of species in an ecosystem. We calculated the importance value of each plant in the sample plot using the formula: importance value = (relative density + relative cover + relative height + relative frequency)/4, and then calculated the above species diversity indices using the R software “vegan” package based on the importance of species. Faith's PD index was used to assess community PD (Faith 1992). Faith's PD represents the minimum total branch length on the phylogenetic tree. The larger its value, the higher the PD. The mean pairwise distance (MPD) of taxa and the mean distance to the nearest taxa (MNTD) were calculated to assess phylogenetic structure (Webb et al. 2002; Di Musciano et al. 2024). MNTD is the weighted average of the phylogenetic distances between each taxon and its closest relative in the community, and MPD is the abundance‐weighted average of the average pairwise phylogenetic distances between taxa within the community. The higher the MPD and MNTD values, the higher the phylogenetic dispersion. To further determine the community's structural patterns and driving mechanisms, we also calculated the net relatedness index (NRI) and net nearest taxa index (NTI) measures. NRI and NTI are obtained by standardizing the observed MPD and MNTD by comparing them with the null model expectation. The NRI index focuses on the similarity between species, while the NTI focuses on the impact between similar species (Chen et al. 2021). The NRI and NTI calculation formulas are as follows:

(1) $$\mathrm{NRI}=-\frac{{\mathrm{MPD}}_{s}-{\mathrm{MPD}}_{\mathrm{mds}}}{\mathrm{SD}\left({\mathrm{MPD}}_{\mathrm{mds}}\right)}$$

(2) $$\begin{array}{c} \mathrm{NTI}=-\frac{{\text{MNTD}}_{s}-{\text{MNTD}}_{\mathrm{mds}}}{\mathrm{SD}\left({\mathrm{MPD}}_{\mathrm{mds}}\right)} \end{array}$$

Among them, SD refers to standard deviation, MPD_s_ and MNTD_s_ represent the actual observed MPD value and the average phylogenetic distance between the most closely related species in the community, and MPD_mds_ and MNTD_mds_ represent the average phylogenetic distance and the average value of the nearest neighbor phylogenetic distance after 999 random simulations by the software. When NRI > 0 and NTI > 0, the phylogenetic structure is clustered; when NRI < 0 and NTI < 0, the phylogenetic structure tends to be dispersed; if NRI = 0 and NTI = 0, it means that the phylogenetic structure of this community is random and the neutral theory plays a dominant role (Zhao et al. 2023). We first determined the species names and classifications using the APG III classification system, constructed a phylogenetic tree using the “V.PhyloMaker2” package, and finally calculated the above indicators based on the phylogenetic tree using the “picante” package.

### 2.5 Data Analysis

Microsoft Excel 2019 was used to preliminarily organize the raw data. Shapiro–Wilk normality test and variance homogeneity test were performed before all data analysis. If there were data that did not conform to the normal distribution, logarithmic transformation (lg(*x*)) was performed. We used linear regression and quadratic regression to explore the spatial distribution patterns of diversity along longitude, latitude and altitude, and selected the best fitting model according to the Akaike Information Criterion (AIC). In order to eliminate the influence of dimensions between variables, we performed *Z*‐score standardization on all data. Pearson correlation analysis was used to explore the relationship between species and PD indicators. In order to analyze the spatial distribution pattern of desert plant community diversity, this study comprehensively considered the multidimensional environmental factors of geography, climate and soil, and conducted a systematic analysis of the spatial distribution pattern of desert plant community diversity. To avoid multicollinearity between variables, we conducted Pearson correlation analysis between environmental factors and eliminated variables with high correlation values (Pearson's |*r*| > 0.8) (Wani et al. 2025). Finally, one geographical factor (longitude), three climatic factors (mean annual temperature [MAT], minimum temperature of the coldest month [MTCM], and annual precipitation [MAP]) and six soil factors (soil organic carbon [SOC], soil available phosphorus [SAP], soil total phosphorus [STP], soil available nitrogen [SAN], soil total nitrogen [STN], and soil pH [SpH]) were retained for subsequent analysis. CANOCO v5.0 software was used to perform redundancy analysis (RDA) to preliminarily explore the effects of environmental factors on community diversity. To further identify the main driving factors, we used diversity indices and environmental variables to construct a multivariate linear regression model. We constructed all possible models using the “MuMIn” package in R software. The model with the minimum AICc value was considered the best fitting model. We also performed collinearity diagnostics for each best model. The variance inflation factor (VIF) was less than 10 for all predictor variables, indicating no serious multicollinearity issues. Finally, the “rdacca.hp” package was used to hierarchically split the influencing factors in each model to clarify the relative importance of each factor. Except for RDA, all the above analyses were performed in R software (version 4.3.2).

## 3 Results

### 3.1 Species Composition of Desert Plant Communities

The plant community structure in the Hexi Corridor is simple, with a small number of species, and each plot consists of 2–7 species. In this study, a total of 39 species of plants belonging to 31 genera and 13 families were investigated, of which Poaceae and Amaranthaceae were the main groups, with Poaceae accounting for 33.59% of the total species and Amaranthaceae accounting for 33.39% of the total species (Figure A1). In addition, we observed that the dominant species in the northwest of the Hexi Corridor were *Hexinia polydichotoma*, *Lycium ruthenicum*, *Phragmites communis* , and *Grubovia dasyphylla*. The main dominant species in the southeast were *Krascheninnikovia ceratoides* , *Asterothamnus alyssoides*, *Sympegma regelii*, and *Leymus chinensis*. The main dominant species in the central part of the Hexi Corridor were *Grubovia dasyphylla*, *Leymus chinensis*, *Artemisia blepharolepis*, *Reaumuria songarica*, *Agropyron cristatum* , *Calligonum mongolicum*, and *Salsola tragus* . The phylogenetic tree of community species is shown in Figure A2.

### 3.2 Geographical Distribution Patterns of Diversity

**FIGURE 3:**
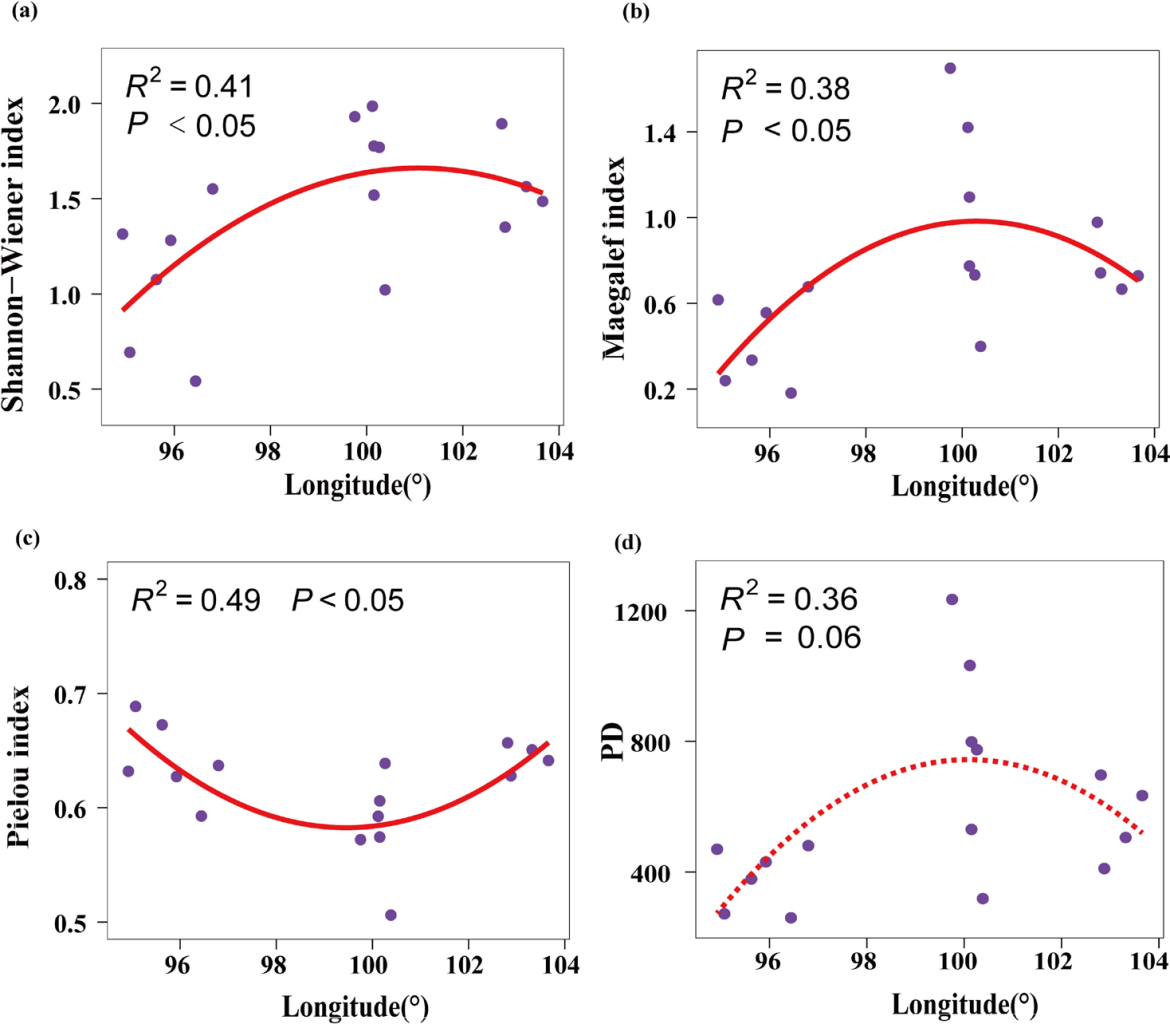
Relationship between Shannon–Weiner diversity index (a), Margalef richness index (b), Pielou evenness index (c), PD index (d) and longitude. The purple dots represent observations from 16 sample strips. The red line indicates the best regression model with the lowest red pool information criterion value. When the red line is dotted, it means the model is not significant.

**FIGURE 4:**
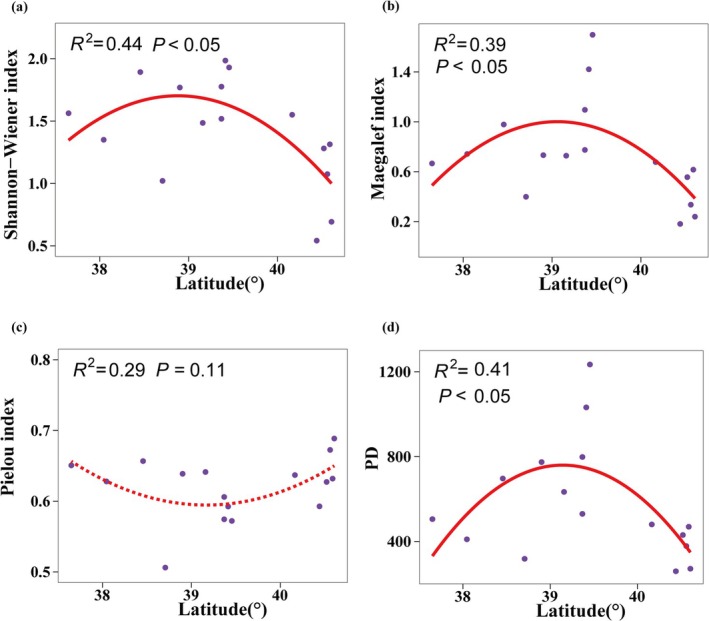
Relationship between Shannon–Weiner diversity index (a), Margalef richness index (b), Pielou evenness index (c), PD index (d) and latitude. The purple dots represent the actual observation data of 16 sample strips. The red line represents the best regression model with the lowest Akaike information criterion value. When the red line is dotted, it means the model is not significant.

The species and PD of desert plant communities showed certain changes along the longitude and latitude gradient. The relationship between the Shannon–Weiner index and the Margalef richness index and longitude and latitude can be described by a quadratic regression model, both showing a hump pattern of first increasing and then decreasing with increasing longitude and latitude (Figures 3a,b and 4a,b). A peak was observed at approximately longitude 100° and latitude 39°. It is worth noting that the Pielou evenness index showed a significant trend of first decreasing and then increasing with increasing longitude (*p* < 0.01) (Figure 3c). The Faith's PD index also showed a significant hump model with latitude (*p* < 0.05) (Figure 4c). No obvious changes in these indices along the altitude gradient were observed.

**FIGURE 5:**
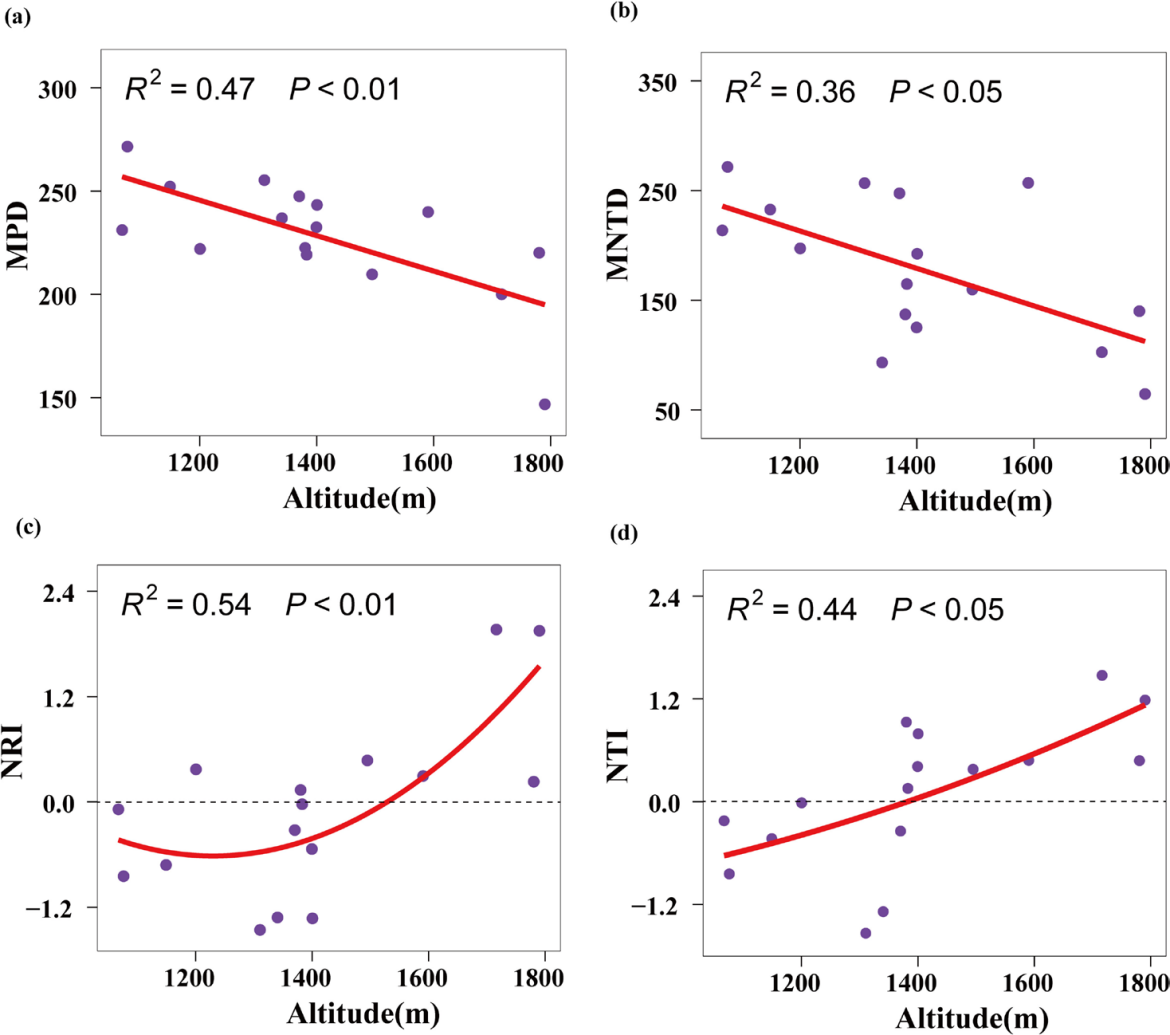
Relationship between MPD index (a), MNTD index (b), NRI index (c), NTI index (d) and altitude. The purple dots represent the actual observation data of 16 sample strips. The red line represents the best regression model with the lowest Akaike information criterion value. When the red line is dotted, it means the model is not significant.

The phylogenetic structure showed certain changes along the altitude gradient. Both the MNTD and MPD indices decreased significantly with increasing altitude (*p* < 0.05) (Figure 5a,b), indicating that the aggregation of high‐altitude communities is stronger than that of low‐altitude communities. The NRI index first decreased and then increased with the altitude gradient (*p* < 0.01) (Figure 5c), and the NTI index increased with increasing altitude (*p* < 0.01) (Figure 5d). Both indices changed from negative to positive values, indicating that with increasing altitude, the phylogenetic structure of the community changed from divergent to aggregated. In addition, the phylogenetic structure did not change significantly along the longitude and latitude gradient.

### 3.3 Relationship Between Species Diversity and Phylogenetic Diversity

**FIGURE 6:**
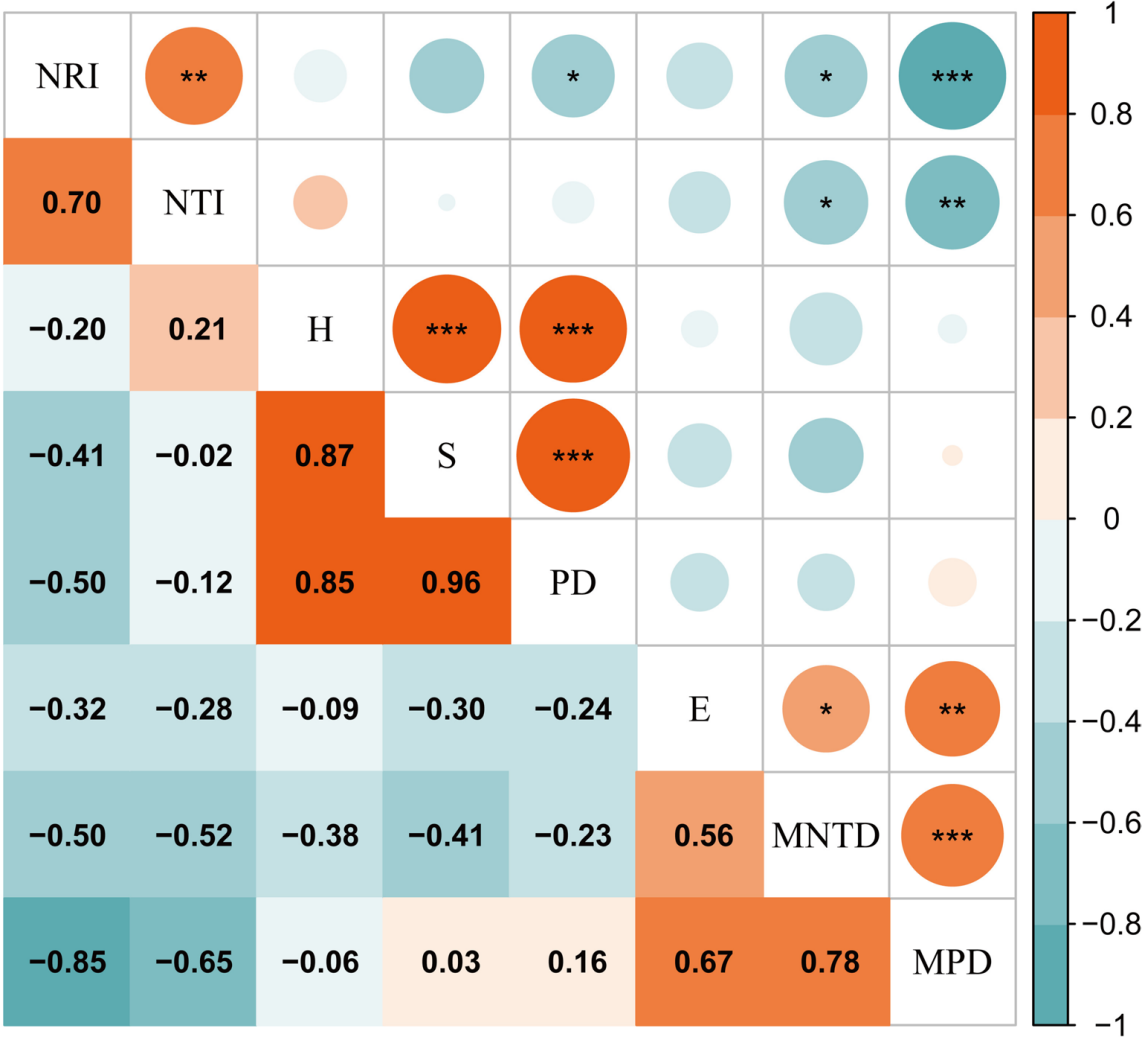
Pearson correlation between species diversity index and phylogenetic index of desert plant communities. *p < 0.05; **p < 0.01; ***p < 0.001. Orange denotes positive correlation, blue denotes negative correlation; the darker the color, the larger the circle, and the larger the absolute value of the number, the stronger the correlation. *E*, Pielou evenness index; *H*, Shannon–Wiener diversity index; MNTD, mean nearest taxonomic unit distance index; MPD, mean pairwise distance index; NRI, NET nearest taxa index; NTI, net nearest taxa index; PD, phylogenetic diversity index; *S*, Margalef richness index.

Pearson correlation analysis showed that species diversity was closely related to PD (Figure 6). The Shannon–Wiener diversity index and Margalef richness index were all positively correlated with the PD index (*p* < 0.001), while the Pielou index was positively correlated with the MNTD index (*p* < 0.05) and with the MPD index (*p* < 0.01). The Shannon–Wiener and Margalef indices were highly synergistic (*p* < 0.001) among the species diversity indices, while there was no significant correlation with the Pielou index. In PD, the MNTD index showed a highly significant positive correlation with the MPD index (*p* < 0.001), and both indices had no significant correlation with the PD index, the Shannon–Wiener index, and the Margalef index. In addition, the NRI and NTI indices showed a significant negative correlation with the MPD and MNTD indices (*p* < 0.05).

### 3.4 Analysis of Driving Factors

**FIGURE 7:**
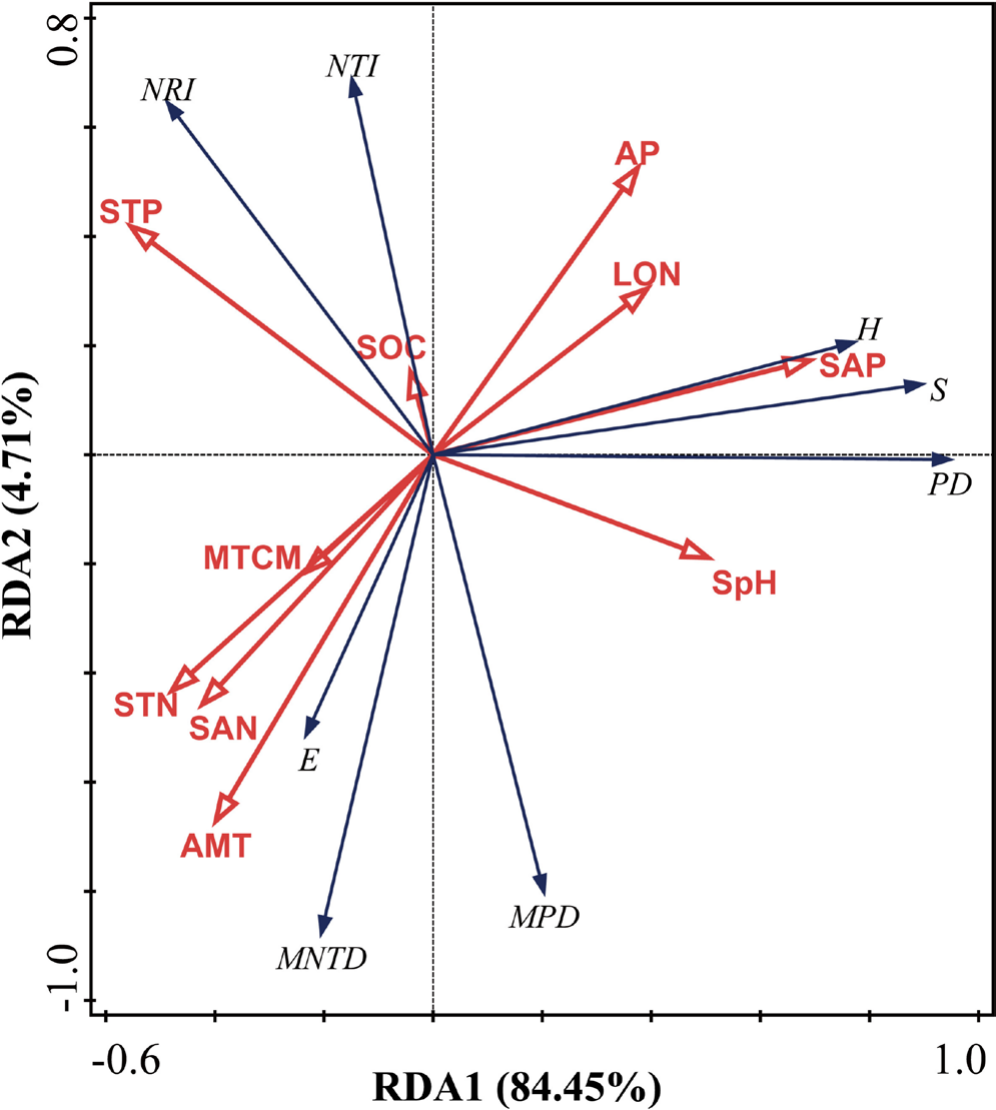
RDA analysis of species diversity index and phylogenetic diversity index with environmental factors. Red lines represent environmental factors. AMT, average annual temperature; AP, annual precipitation; Lon, longitude; MTCM, minimum temperature of the coldest month; SAN, soil available nitrogen; SAP, soil available phosphorus; SOC, soil organic carbon; SpH, soil pH; STN, soil total nitrogen; STP, soil total phosphorus.

The results of redundancy analysis (Figure 7) showed that the first axis explained 84.45% of the variation, and the second axis explained 4.71%. The two axes together explained 89.61% of the variation in species and PD, which can better reflect the relationship between community diversity and environmental factors. Among them, the Shannon–Wiener, Margalef, and PD indexes have a small angle with soil available phosphorus, so these indicators show a strong positive correlation with soil available phosphorus and have a large obtuse angle with soil total phosphorus, soil total nitrogen, and annual average temperature, which may be negatively correlated with these environmental factors. Similarly, the Pielou index shows a strong positive correlation with soil total nitrogen, the lowest temperature of the coldest month, and the annual average temperature. In addition, the MNTD and MPD indices show a strong positive correlation with the annual average temperature and may be negatively correlated with environmental factors such as precipitation and soil total phosphorus. The NRI and NRI index have a large angle with the annual average temperature, soil pH, and soil total nitrogen, showing a strong negative correlation, but may show a positive correlation with soil total phosphorus.

**FIGURE 8:**
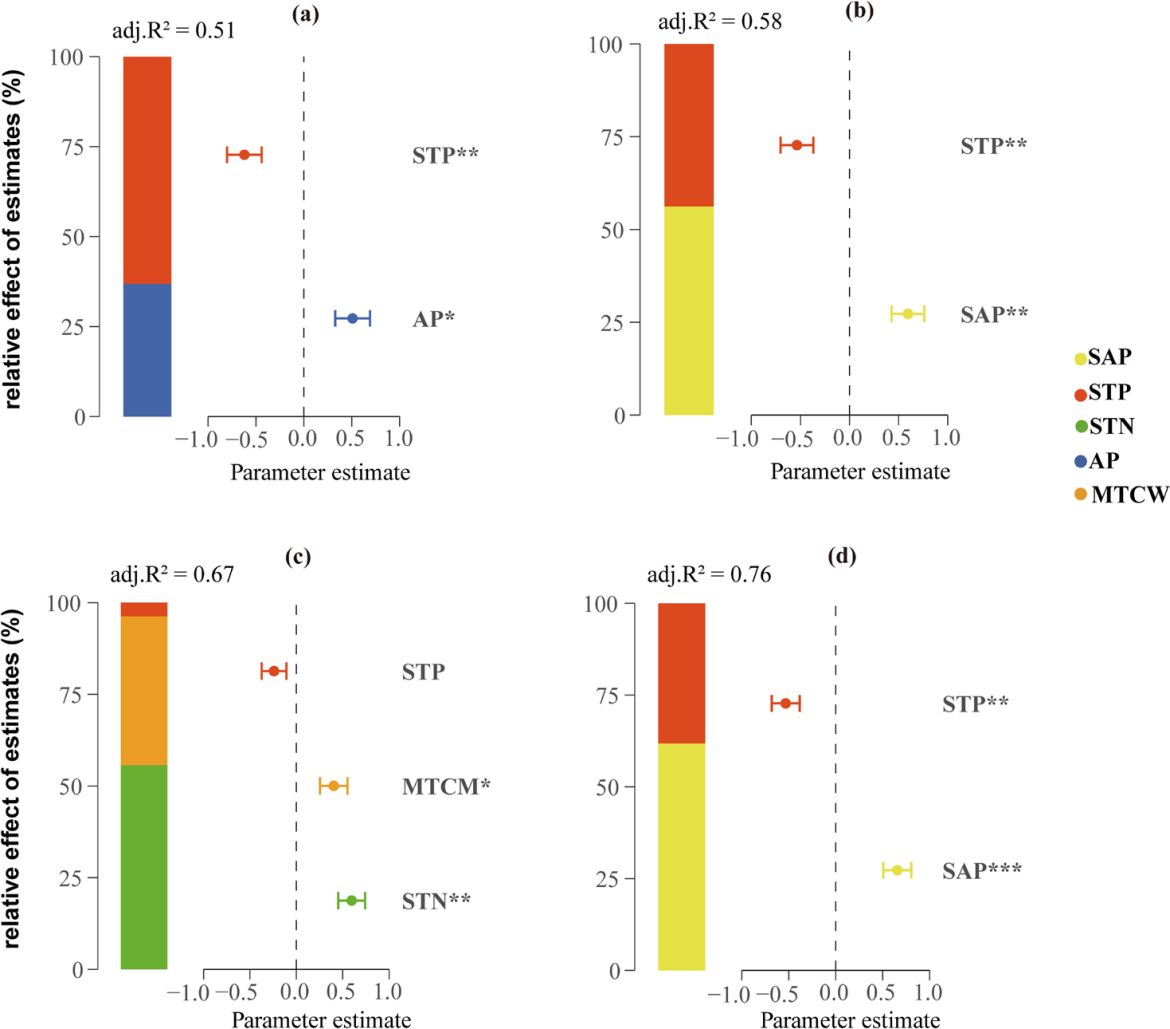
Relative effects of environmental factors on species diversity ([a] Shannon–Wiener, [b] Margalef, [c] Pielou) and phylogenetic diversity ([d] PD). The average parameter estimates (standardized regression coefficients) of the model predictors (environmental variables) and their 95% confidence intervals, as well as the relative importance of each predictor, are expressed as a percentage of explained variance, and the model‐adjusted *R* ^2^ is shown. Statistical significance is marked as: *p < 0.05; **p < 0.01; ***p < 0.001.

**FIGURE 9:**
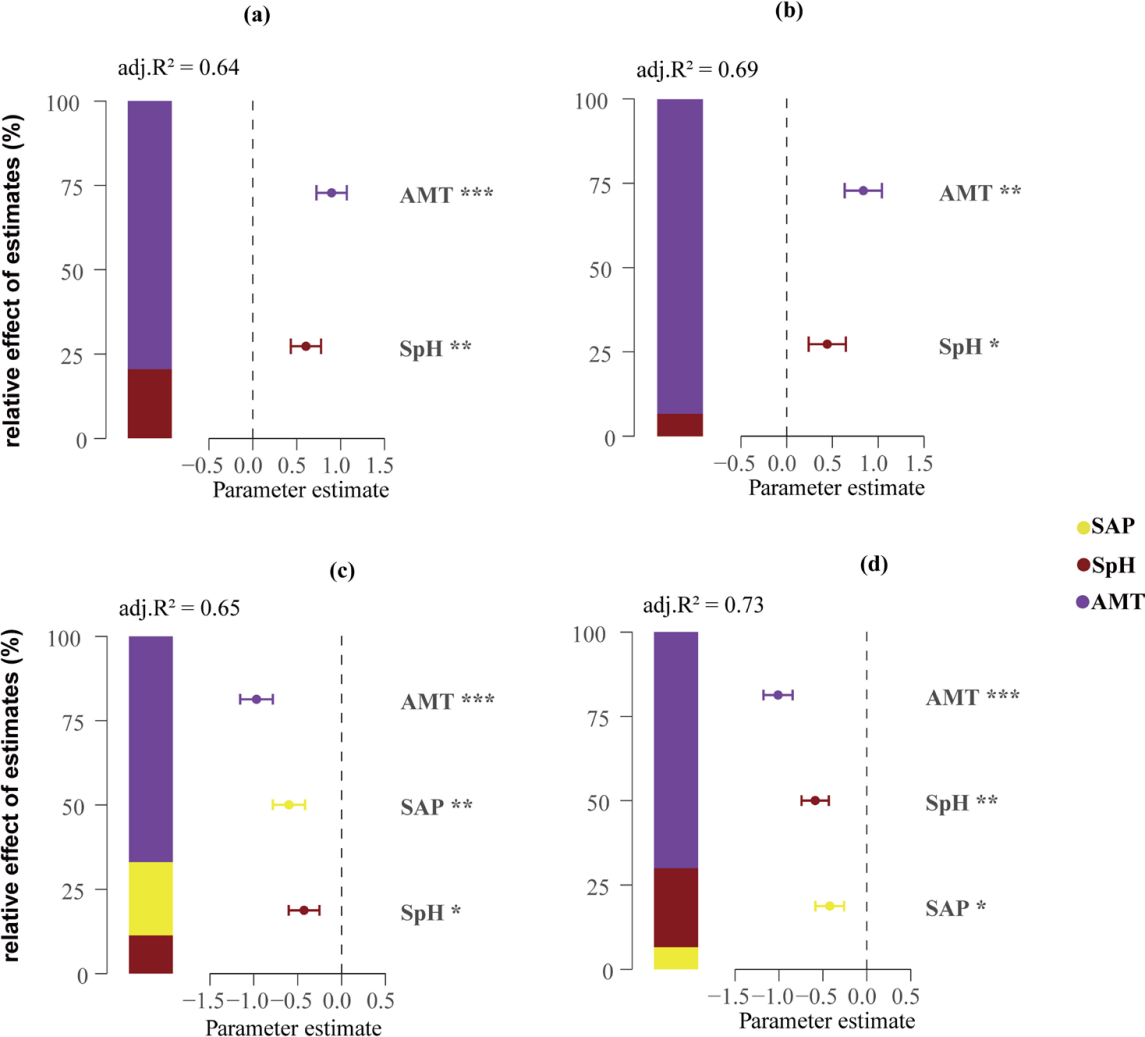
Relative effects of environmental factors on phylogenetic structure ([a] MPD, [b] MNTD, [c] NRI, [d] NTI). The average parameter estimates (standardized regression coefficients) of the model predictors (environmental variables) and their 95% confidence intervals, as well as the relative importance of each predictor, are expressed as a percentage of explained variance, and the model‐adjusted *R* ^2^ is shown. Statistical significance is marked as: *p < 0.05; **p < 0.01; ***p < 0.001.

Multiple regression analysis was used to further determine the dominant factors of species and PD. The results showed that soil environmental variables were the main influencing factors for species diversity. Among them, soil total phosphorus was extremely significantly negatively correlated with the Shannon–Wiener index (*p* < 0.01), explaining 63% of its total variation (Figure 8a); soil available phosphorus was extremely significantly positively correlated with the Margalef index (*p* < 0.01), explaining 56% of its total variation (Figure 8b); soil total nitrogen was significantly positively correlated with the Pielou index (*p* < 0.01), explaining 56% of its total variation (Figure 8c). For PD, soil available phosphorus was extremely significantly positively correlated with the PD index (*p* < 0.001) and explained 62% of the total variation of the PD index, which was the main influencing factor (Figure 8d). The annual average temperature showed a very significant positive correlation with the MNTD index and the MPD index (*p* < 0.001), explaining 79% of the total variation of the MNTD index and 93% of the total variation of the MPD index, respectively, and dominating the changes of these two indicators (Figure 9a,b). The driving factor of NRI and NTI is also the annual mean temperature. It can be observed that the annual mean temperature explains 69% of the total variation of the NRI index (Figure 9c) and 75% of the total variation of the NTI index (Figure 9d).

## 4 Discussion

Our study revealed the spatial variation patterns of species diversity along the latitudinal and longitudinal gradients of the Hexi Corridor. Both the Shannon–Wiener index and Margalef index exhibit a unimodal pattern (increasing then decreasing) across these gradients (Figures 4 and 5), a pattern also documented in other arid regions (Li et al. 2020). However, this contrasts sharply with findings from previous studies encompassing the broader northwestern region, which reported an initial decrease followed by an increase in species diversity along similar gradients (Jianming et al. 2017). This discrepancy underscores how plant diversity distribution patterns vary significantly with study region and scale. We identified significant correlations between both latitude/longitude and annual precipitation (Figure A3). In arid zones, increased precipitation facilitates plant photosynthesis and respiration, improves soil ecological conditions, and promotes the growth of less drought‐tolerant species (Yang, Li, et al. 2019; Huxman et al. 2004). However, abundant water resources can also intensify interspecific competition, potentially leading to a reduction in species richness (Adler and Levine 2007). Furthermore, previous research indicates that spatial diversity patterns are driven by environmental heterogeneity and represent the combined effects of multiple ecological factors (Stein et al. 2014). The southeastern Hexi Corridor experiences relatively higher precipitation but is constrained by limited heat availability, while the northwestern region faces intensified heat and drought stress. The central area, however, benefits from a resource optimization window created by topography‐mediated water–heat balance. Our analysis further identifies available phosphorus (SAP) as a key driver of the species diversity distribution pattern (Figure 8). SAP concentrations are notably higher in the central region (Figure A4), a distribution likely attributed to the combined influence of vegetation, climate, and topography (Shao et al. 2022). Consequently, the optimal soil, water, and climatic conditions in this central zone create a balance of resources and stressors, supporting the coexistence of diverse plant species and resulting in the observed diversity peak. PD also displayed a significant hump‐shaped pattern along the latitudinal gradient. We found a highly significant positive correlation between PD and species richness indices (Figure 6), suggesting the PD hump pattern may be driven by the corresponding pattern in species richness. The suitable environmental conditions in the central Hexi Corridor support species unable to survive in the extreme environments of the southeast or northwest, thereby fostering higher PD in this region. In summary, conservation strategies should prioritize the desert plant communities in the central Hexi Corridor.

Interestingly, the evenness index shows a trend of first decreasing and then increasing with increasing longitude, which is opposite to the longitude variation pattern of the Shannon–Wiener index and the Margalef index. Compared with other species diversity indices, the Pielou index reflects another aspect of community complexity, focusing on describing the distribution of the number of individuals of each species in the community (Wilsey and Potvin 2000). As the richness increases, the dominance of dominant species increases, while the dominance of rare species decreases (Cheng et al. 2021). This gap decreases as the species richness decreases, so the community in the central region shows a lower evenness. In addition, we did not find a clear pattern of change in species diversity along the altitude gradient, which is different from the commonly observed patterns that species diversity decreases monotonically along the altitude gradient or expands at the central altitude (Dong et al. 2019). This study did not set a strict altitude gradient sequence, and the limited altitude range investigated and the insufficient sample point layout may have led to this result. In addition, the key environmental factors that affect diversity have a small change range or lack obvious patterns along the altitude gradient, which may also lead to the fact that the distribution of species diversity does not show a significant change pattern along the altitude gradient. Therefore, future studies should increase the sample point layout within a larger altitude gradient range to further explore the variation pattern of diversity.

We found that soil nutrients are key drivers of spatial distribution patterns of species diversity and PD. Although climate is often considered a key factor in shaping the spatial pattern of plant diversity in large‐scale analyses (Wang et al. 2011), this relationship shows significant scale dependence, resulting in changes in the driving mechanism of diversity formation with changes in geographic scale (Tang et al. 2012). Compared with climate factors, soil nutrients directly affect plant growth, development, and spatial distribution (Yang, Chen, and Yang 2019). Soil spatial heterogeneity can promote plant species coexistence and diversity by increasing resource diversity and providing habitat shelter (Sellan et al. 2019). This study found that soil available phosphorus was significantly positively correlated with the Margalef index and PD index and was the most important influencing factor. This view is consistent with other studies that believe that available phosphorus is one of the key limiting factors in desert ecosystems (Sun et al. 2023), indicating that phosphorus availability often limits plant growth (Grinsted et al. 1982). Soil available energy directly drives the improvement of plant diversity by promoting plant growth and root activity, while the increase in plant diversity increases the availability of soil environmental phosphorus through accelerated litter decomposition, microbial‐mediated phosphorus activation, and enhanced phosphorus cycle, forming a positive feedback mechanism to jointly maintain ecosystem productivity and species richness (Dong et al. 2025). In addition, the increase in species richness further increased the PD of the community. This study also found that soil total phosphorus was negatively correlated with the Shannon–Wiener index, which can be explained by the fact that in arid areas, due to the large amount of calcium salts in the soil, high total phosphorus may reflect an increase in the fixed form of phosphorus rather than bioavailable phosphorus (Zhang, Shi, et al. 2020), which is difficult for plants to directly absorb. In addition, some studies have pointed out that in some arid or semi‐arid areas, areas with accumulated total phosphorus may also show high salinity and high pH values. This environment is not conducive to plant growth, thereby inhibiting plant diversity (Wang et al. 2023). Of course, this may also be caused by uneven nutrient distribution. Soil total nitrogen is the main influencing factor of the Pielou evenness index and is positively correlated with it. Previous studies have shown that an increase in nitrogen content in nitrogen‐limited areas may alleviate resource competition pressure and promote species diversity (Suding et al. 2004). The increase in evenness may be related to the special geographical distribution of nitrogen. We found that nitrogen showed a pattern of first decreasing and then increasing along the longitude and latitude gradient (Figure A4). This distribution pattern reduces the monopoly of dominant species on resources and gives more species the opportunity to survive. This study also found that evenness is significantly positively correlated with the minimum temperature of the coldest month. Low temperature induces plant roots to secrete more sugars, attracting mycorrhizal fungi to colonize and form a hyphal network. This network can connect the roots of different plants and promote the transfer of nutrients (such as water, nitrogen, and phosphorus) between species (Umar et al. 2024). At the same time, the microenvironment formed by the plants themselves under low temperatures has a significant buffering effect and can provide more stable living conditions for neighboring species (Cavieres et al. 2007). These mechanisms enhance the promotion of plants in extreme environments, optimize resource allocation among species, and thus improve community evenness. In summary, the effects of soil nutrients on plant diversity are complex, and the effects of different nutrient forms on diversity reveal the dynamic balance between resource limitation and species adaptation in arid ecosystems.

Studying phylogenetic structure helps to understand the assembly mechanism of plant communities. Our results show that as the altitude increases, the degree of dispersion of desert plant communities becomes lower, and the structure changes from divergence to aggregation. The communities investigated in this study have a certain phylogenetic structure, and no randomness caused by neutral processes was observed. Most studies attribute this pattern to the fact that the low‐altitude environment is suitable, so competitive exclusion between plants is dominant, and the high‐altitude environment is harsh, and environmental filtering plays a major role (Manish and Pandit 2018; Li et al. 2014). In the Hexi Corridor, altitude is extremely significantly positively correlated with annual precipitation and extremely significantly negatively correlated with temperature (Figure A3). Low‐altitude environments are often accompanied by high temperatures and high evapotranspiration, low precipitation, and high drought levels. This relatively harsh environment community structure still shows a divergent trend. We speculate that the drought adaptation strategy of desert plants is not very conservative in phylogeny. Plants have evolved for a long time under strong pressure, so that distant species show evolutionary convergence and form a variety of complex drought resistance strategies (Chaves et al. 2021). Second, although there is strong competition among plants under extreme conditions (Tu et al. 2024), the promotion effect between plants is stronger, and some species can act as collaborators with distant relatives to cope with extreme environments (Massante et al. 2021). This high divergence trend under high environmental pressure is also similar in other studies (Qian et al. 2014). However, at high altitude and low aridity, the community structure divergence was low and the community results were clustered, which may be due to the fact that competitive exclusion between plants is related to the entire branch (Mayfield and Levine 2010), rather than only occurring between close relatives with overlapping niches. Closely related species are more competitive than distantly related species, resulting in lower community dispersion. It may also be due to the high trait differences between closely related lineages (Prinzing et al. 2008), which promotes resource utilization segmentation. This approach reflects the balance mechanism between plants. Soil pH also affects phylogenetic structure. The possible reason is that the alkaline soil environment in desert areas reduces soil nutrients, further restricts resources, and reduces the stability of dominant species, allowing distantly related species to coexist (Liu et al. 2022). Future in‐depth research on the phylogenetic structure of the Hexi Corridor should focus on climate factors, but soil factors should not be ignored, and further analysis in combination with the characteristics of desert plants is particularly important.

This study has revealed the distribution pattern of plant diversity in the Hexi Corridor to a certain extent, but it also has certain limitations. We established 16 representative sample plots in the region. However, although spatial representativeness was taken into account when selecting the sample plots, the current sample size is still slightly insufficient due to the uneven distribution and strong spatial heterogeneity of desert plants, which may limit the comprehensive characterization of regional plant diversity. Future studies can expand on this basis and increase the number and coverage of sample plots to more comprehensively and accurately illustrate the vegetation diversity pattern in the region. In addition, since the spatial distribution of plants is also affected by the covariation and interaction of multiple environmental factors (López‐Angulo et al. 2020; Li et al. 2022), this study has not yet explored this issue in depth, so further analysis is needed on the interactive effects among environmental factors in the Hexi Corridor and their specific effects on distribution patterns.

## 5 Conclusion

In summary, this study shows that species richness and PD show a hump model with longitude and latitude. Therefore, in the Hexi Corridor, the central region is the best condition for the growth and distribution of desert plants, and the central region can be given priority in formulating protection strategies. The study also shows that environmental factors such as soil available phosphorus, total nitrogen, total phosphorus, and average annual temperature have a significant impact on community diversity. Soil factors, especially available phosphorus, have a particularly prominent positive effect on diversity, while the regulatory effect of average annual temperature on phylogenetic structure is more obvious. This result emphasizes the importance of comprehensively considering climate and soil factors in the protection of desert ecosystems. In addition, this study found that the phylogenetic structure is over‐dispersed at low altitudes and more aggregated in high altitude areas, indicating that desert plants may have multiple ways to achieve drought resistance. However, the formation mechanism of phylogenetic structure still needs further study, especially focusing on the conservation of key functional traits of plants. This study deepens our understanding of the formation mechanism of community diversity in the desert ecosystem of the Hexi Corridor and provides a theoretical basis for the formulation of effective protection strategies.

## Author Contributions

**Xinyi Zhou:** investigation (equal), methodology (equal), validation (equal), visualization (equal), writing – original draft (lead), writing – review and editing (equal). **Zhaoxiang Zhang:** investigation (equal), methodology (equal), validation (equal), visualization (equal), writing – review and editing (equal). **James F. White:** methodology (equal), validation (equal), visualization (equal), writing – review and editing (equal). **Yingxiang Miao:** investigation (equal), methodology (equal), writing – review and editing (equal). **Shanjia Li:** conceptualization (lead), funding acquisition (lead), supervision (lead), writing – review and editing (equal).

## Conflicts of Interest

The authors declare no conflicts of interest.

## Supporting information


**Figure A1.** Abundance of frequent plant families among 39 species from communities. Percentage is the proportion of all species in the same family to the total number of species.

**Figure A2.** Phylogenetic tree of desert plants in the Hexi Corridor.

**Figure A3.** Pearson correlation between environmental factors. *p < 0.05; **p < 0.01; ***p < 0.001. Red indicates positive correlation, blue indicates negative correlation; the darker the color, the larger the circle, and the larger the absolute value of the number, the stronger the correlation. Alt, altitude; AMT, average annual temperature; AP, annual precipitation; Lat, latitude; Lon, longitude; MTCM, minimum temperature of the coldest month; MTWM, maximum temperature of the warmest month; SAN, soil available nitrogen; SAP, soil available phosphorus; SOC, soil organic carbon; SpH, soil pH; STN, soil total nitrogen; STP, soil total phosphorus.

**Figure A4.** Spatial distribution patterns of key soil factors. (a) and (b) represent the changing trends of soil available phosphorus and soil total phosphorus in longitude, respectively; (c) and (d) represent the changing trends of soil available phosphorus and soil total phosphorus in latitude, respectively. The purple dots represent the actual observation data of 16 sample strips. The red line represents the best regression model with the lowest Akaike information criterion value.

**Data S1:** ece372114‐sup‐0005‐Supinfo.xlsx.

## Data Availability

The datasets used in this study are available in the Supporting Information.
