# Supplementary figures and images for: Geographical Patterns and Drivers of Species and Phylogenetic Diversity of Desert Plant Communities in the Hexi Corridor, Northwestern China
Source: Ecol Evol. 2025 Sep 7;15(9):e72114. doi: 10.1002/ece3.72114 (PMC12414604; doi:10.1002/ece3.72114)

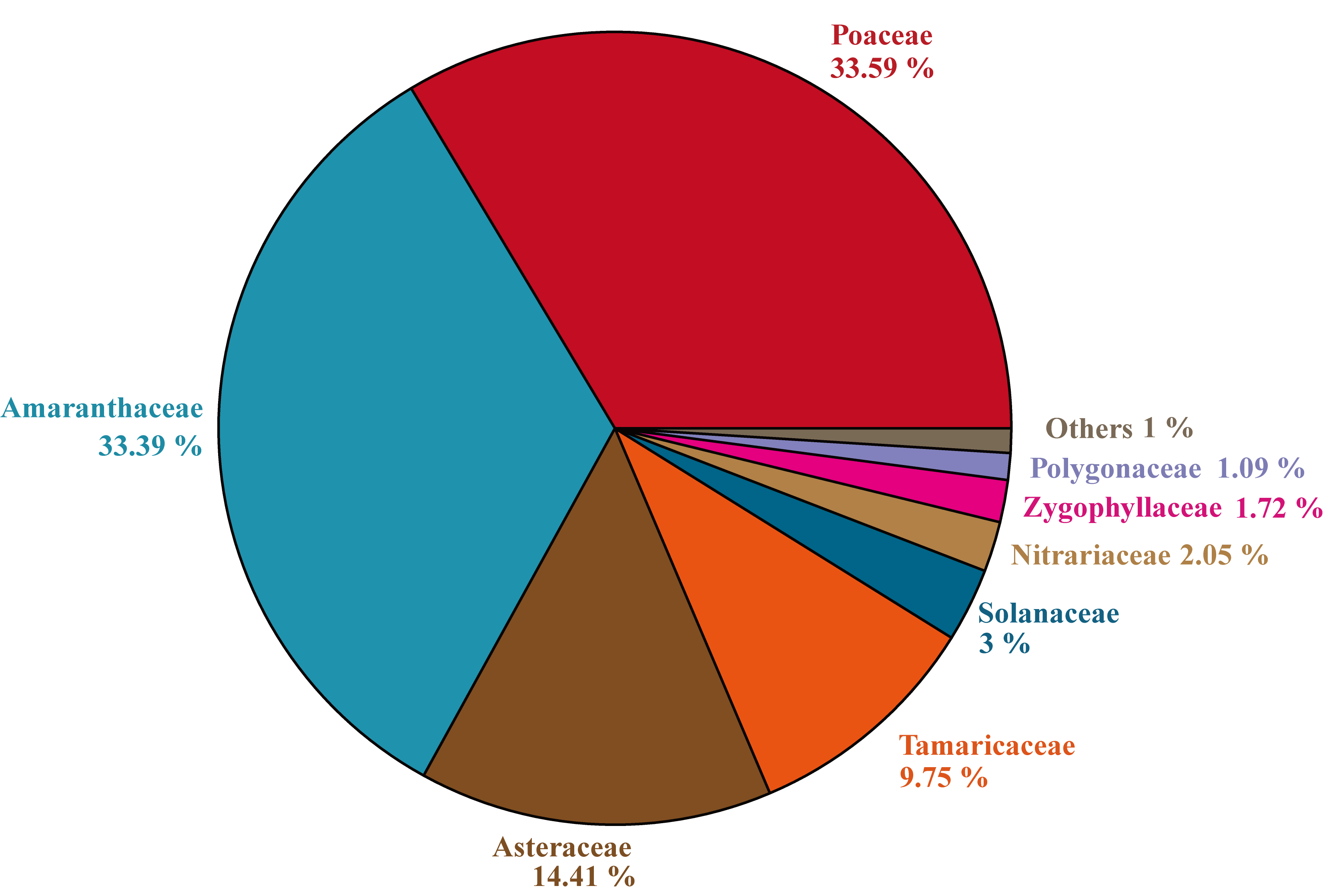

Supplement: Supplementary file 1 — Figure A1. Abundance of frequent plant families among 39 species from communities. Percentage is the proportion of all species in the same family to the total number of species. [file ECE3-15-e72114-s001.tif]

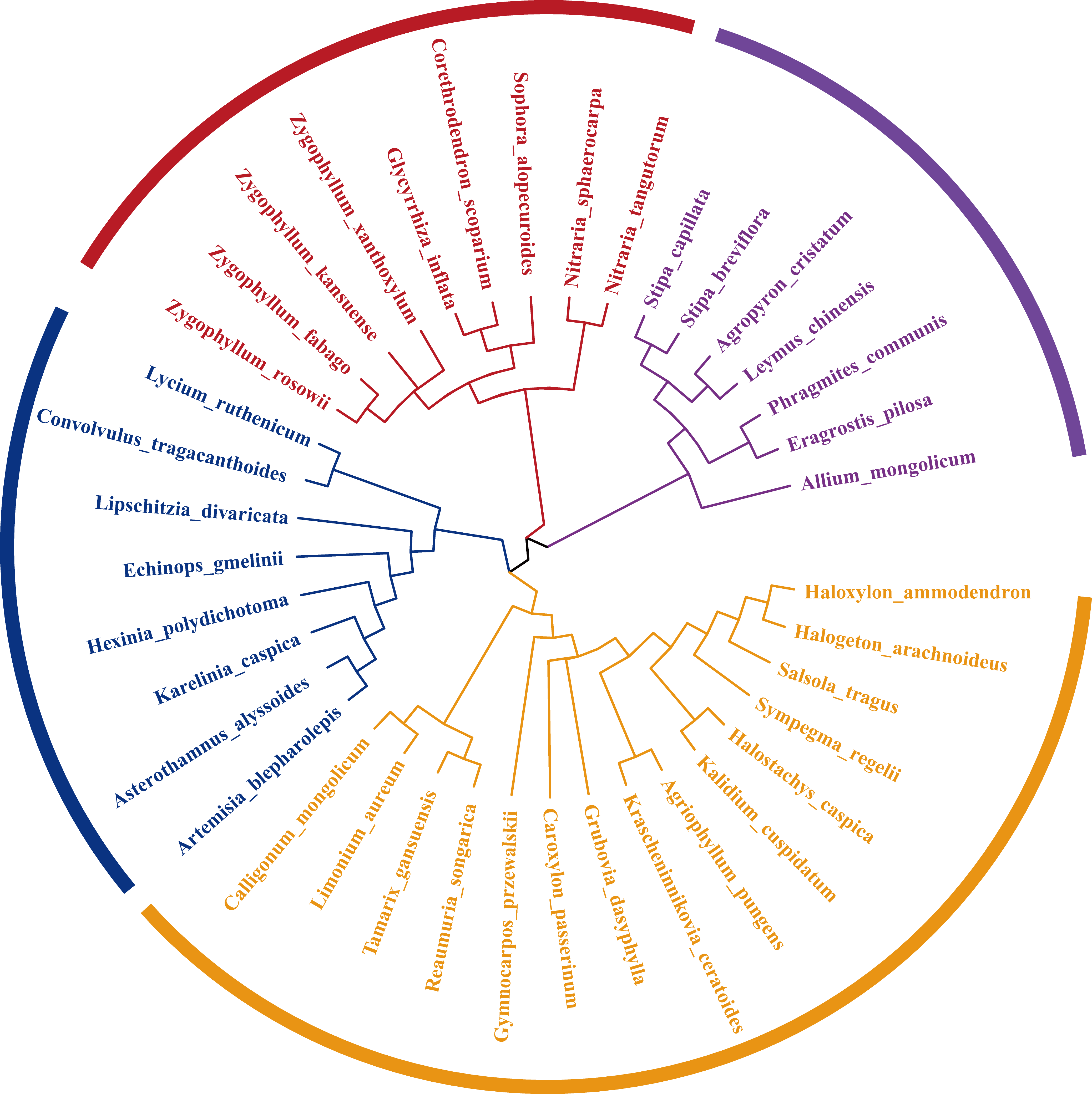

Supplement: Supplementary file 2 — Figure A2. Phylogenetic tree of desert plants in the Hexi Corridor. [file ECE3-15-e72114-s002.tif]

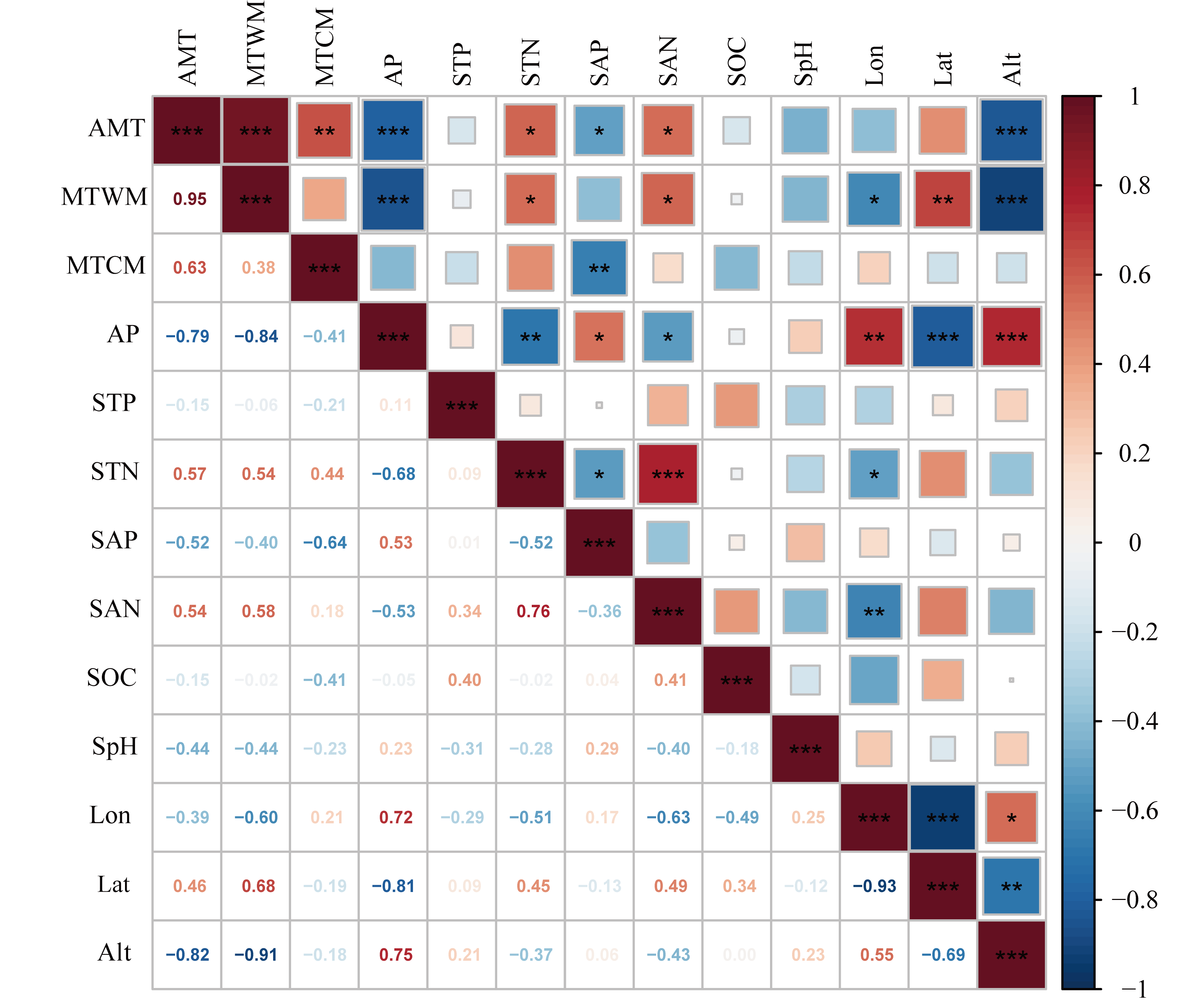

Supplement: Supplementary file 3 — Figure A3. Pearson correlation between environmental factors. *p < 0.05; **p < 0.01; ***p < 0.001. Red indicates positive correlation, blue indicates negative correlation; the darker the color, the larger the circle, and the larger the absolute value of the number, the stronger the correlation. Alt, altitude; AMT, average annual temperature; AP, annual precipitation; Lat, latitude; Lon, longitude; MTCM, minimum temperature of the coldest month; MTWM, maximum temperature of the warmest month; SAN, soil available nitrogen; SAP, soil available phosphorus; SOC, soil organic carbon; SpH, soil pH; STN, soil total nitrogen; STP, soil total phosphorus. [file ECE3-15-e72114-s003.tif]

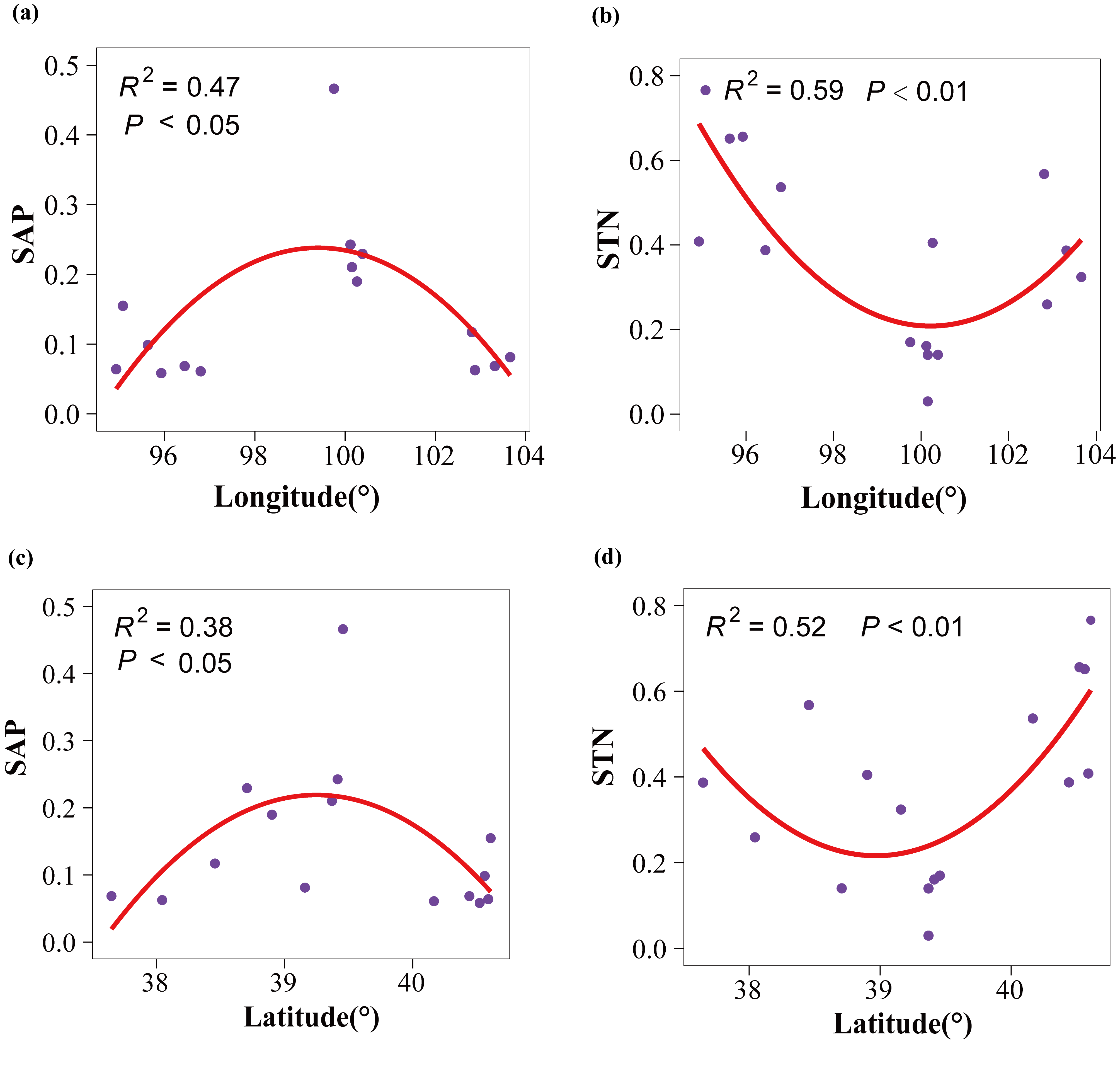

Supplement: Supplementary file 4 — Figure A4. Spatial distribution patterns of key soil factors. (a) and (b) represent the changing trends of soil available phosphorus and soil total phosphorus in longitude, respectively; (c) and (d) represent the changing trends of soil available phosphorus and soil total phosphorus in latitude, respectively. The purple dots represent the actual observation data of 16 sample strips. The red line represents the best regression model with the lowest Akaike information criterion value. [file ECE3-15-e72114-s005.tif]
